# Detection of Islet Autoantibodies in Whole Blood by Antibody Detection by Agglutination-PCR (ADAP) Technology Is Sensitive and Suitable for General Population Screening Programs

**DOI:** 10.1155/2024/4238394

**Published:** 2024-03-14

**Authors:** Tal Oron, Felipe de Jesus Cortez, Biana Shtaif, Peter V. Robinson, Michal Yackobovitch-Gavan, Devangkumar Tandel, David Seftel, Moshe Phillip, Cheng-ting Tsai, Galia Gat-Yablonski

**Affiliations:** ^1^The Jesse Z and Sara Lea Shafer Institute for Endocrinology and Diabetes, National Center for Childhood Diabetes, Schneider Children's Medical Center of Israel, Petach Tikva, Israel; ^2^Faculty of Medicine, Tel Aviv University, Tel Aviv, Israel; ^3^Enable Biosciences Inc., South San Francisco, CA, USA; ^4^Laboratory for Molecular Endocrinology and Diabetes, Felsenstein Medical Research Center, Petach Tikva, Israel; ^5^Department of Epidemiology and Preventive Medicine, School of Public Health, Faculty of Medicine, Tel Aviv University, Tel Aviv, Israel

## Abstract

**Background:**

Detection of type 1 diabetes (T1D) at the preclinical stage is possible by detecting islet autoantibodies (IAs) years before the appearance of symptomatic diabetes. The Antibody Detection Israeli Research is a general population screening program searching for children with multiple IAs who are at risk of developing T1D. IAs are measured in capillary or venous whole blood (WB) samples using the novel ultrasensitive antibody detection by agglutination-PCR (ADAP) technology.

**Objective:**

To assess the accuracy and reliability of the ADAP assay in venous and capillary WB.

**Materials and Methods:**

In total, 50 children with T1D and 50 healthy controls participated in the study. Venous and capillary blood samples were drawn from participants with T1D, while only venous blood was drawn from the controls. The ADAP assay in venous and capillary blood was compared to the currently used assays in their ability to detect glutamic acid decarboxylase (GADA), islet antigen-2 (IA-2A), and insulin autoantibodies (IAAs).

**Results:**

The area under the curve using the receiver operating characteristic curves was comparable between the ADAP assay in WB and standard enzyme-linked immunosorbent assay (ELISA)/radioimmunoassay (RIA) for all three IAs GADA 0.946 (95% CI: 0.900–0.991) vs. 0.949 (0.906–0.992), *P*=0.873; IA-2A 0.747 (0.649–0.844) vs. 0.666 (0.587–0.744), *P*=0.106; IAA 1.000 (1.000–1.000) vs. 1.000 (1.000–1.000), *P*=1.000. The correlation between the levels of IA in venous and capillary WB using ADAP was *R*^2^ = 0.958 (*P*  < 0.01), *R*^2^ = 0.943 (*P*  < 0.01), and *R*^2^ = 0.711 (*P*  < 0.01) for GADA, IA-2A, and IAA, respectively. IA levels in venous and capillary WB using ADAP were comparable without a proportional bias in Bland–Altman's plots of agreement, suggesting the two methods may be used interchangeably.

**Conclusions:**

The ADAP assay is reliable in detecting IA in venous and capillary WB samples with comparable performance to standard RIA and ELISA. These findings open avenues for widespread use of the ADAP assay in future general population screening programs to detect children at risk of developing T1D.

## 1. Introduction

Type 1 diabetes (T1D), usually diagnosed during childhood, results from the destruction of insulin-producing pancreatic *β*-cells [1]. The global incidence of the disease has dramatically increased throughout the last decades [1]. Worldwide, 30%–40% of all newly diagnosed T1D patients are presented with diabetic ketoacidosis (DKA), a life-threatening event associated with long-term sequels, including decreased metabolic control, an increased risk for vascular complications, and memory deficits [2, 3]. To date, early detection of T1D is possible by detecting islet autoantibodies (IAs). About 80% of the children diagnosed with T1D have multiple IAs before the age of 5 years, with an estimated progression rate of about 84% to symptomatic diabetes by 15 years [4]. Diagnosing T1D at the preclinical stage can prevent the occurrence of DKA at the clinical presentation of the disease. It may also open a path for future disease prevention programs [5–7]. An efficient screening program based on IA will identify children at risk of developing diabetes during childhood, enable caregivers to prepare at-risk children and their families for future insulin treatment, prevent DKA episodes upon clinical presentation, and aid in the search for an effective interventional therapy for T1D [7, 8]. The Antibody Detection Israeli Research (ADIR), initiated in October 2021, is a general population screening program in Israel to identify children with multiple IAs at risk of developing T1D [8]. The infrastructure of ADIR includes over 40 community-based screening sites, six diabetes centers, a central laboratory dedicated to the study, and a coordinating center. The ADIR study aims to screen 25,000 children aged 9–18 months all over Israel for IA. IAs are measured in capillary whole blood (WB) using the novel ultrasensitive antibody detection by agglutination-PCR (ADAP) assay by Enable Biosciences^©^ [9, 10]. We have previously shown that the ADAP assay reliably detects the three cardinal IAs, namely, glutamic acid decarboxylase (GADA), islet antigen-2 (IA-2A), and insulin autoantibodies (IAAs) in 20 *µ*L of serum with higher sensitivity than the currently used radio-binding assays (RBA) and radio-immune assays [11, 12]. Nevertheless, the reliability and accuracy of the assay were not tested in venous or capillary WB, which is a more desirable sample matrix for general population screening programs.

As part of the instituting process of the ADIR study, we evaluated the performance of the ADAP assay in capillary and venous WB and compared it to the immunoassays that are currently used. The study's main goal was to validate the ability of the ADAP assay to accurately detect IAs in venous and capillary WB, proving it is suitable for a general population screening program.

## 2. Materials and Methods

A prospective, case–control study evaluating the presence of IA in children with T1D and healthy controls using ADAP, RBA, and radioimmunoassay (RIA).

### 2.1. Study Population

In total, 50 children and young adults with T1D and 50 healthy controls without diabetes, aged 5–30 years, were recruited for the study from our diabetes and endocrine clinic at Schneider Children's Medical Center of Israel. Participants with T1D were included in the study regardless of their IA status at disease diagnosis. Excluded from the study were participants with type 2 diabetes, a known hemophilic disease, and those treated with immunosuppressive drugs, including steroids given at a therapeutic dosage. Our institutional ethics committee approved the study, and all the participants or their legal guardians signed an informed consent.

### 2.2. Study Design and Methods

Venous and capillary blood samples were drawn from participants with T1D, while only venous blood was drawn from the controls. We evaluated the samples for the presence of three IAs, namely, GADA, IA-2A, and IAAs. To study the performance of the ADAP assay in venous WB, we analyzed the venous samples of the patients and controls by the ADAP assay and the currently used RIA or enzyme-linked immunosorbent assay (ELISA). To study the performance of the ADAP assay in capillary blood, comparing it to venous WB, capillary blood taken only from the patients was analyzed for the presence of IA using the ADAP assay. About 200 *µ*L of neat blood without further processing were taken for the ADAP assay, transferred to the ADIR lab on the same day, and kept at −80°C until assayed. 5 mL of blood was separated into serum for the RIA and ELISA and analyzed according to the standard procedures.

### 2.3. The ADAP Assay

The ADAP assay takes advantage of the capacity of antibodies to agglutinate [9]. In short, serum, or WB in this study, is mixed with antigen conjugated to DNA and a “bridge oligo” complementary to both DNA strands. The assay leverages the multivalency of antibodies to aggregate antigen-DNA conjugates into proximity. If antibodies are present, the antigen–DNA conjugates aggregate, driving the ligation of DNA strands and producing a new and distinct PCR reporter, which is amplified and quantified by real-time PCR. Thus, the PCR-amplifiable DNA is only formed upon binding of autoantibodies to their antigens, dramatically reducing background and improving signal. The ADAP assay is distinct from traditional immune assays in that the autoantigen–DNA conjugate probe is fully suspended in the solution phase throughout the assay process, and no washing, centrifugation, or extraction is required for signal measurement. Therefore, the autoantigen can be retained in its native conformation and exposed to all surface epitopes for autoantibody binding. The ADAP T1D assay was independently validated in the International Antibody Standardization Program 2018, performing well for all three IAs [13]. It is a fully automated, multiplex high throughput assay testing for IA in a small amount of capillary blood and, therefore, may be suitable for a general population screening program. The ADAP assay and its advantages were previously reported [12–15]. In the current study, the assay was performed in the ADIR lab, which was founded primarily for the study.

### 2.4. RIA and ELISA Assays

ELISA and RIA assays were done in serum samples. IAAs were determined by the DIAsource IAA RIA Kit (DIAsource ImmunoAssays S.A., Louvain-la-Neuve, Belgium) according to the manufacturer's protocol. IAA levels ≥0.4 U/mL were considered positive. GADA and IA-2A were determined by ElisaRSR™ GADAb and ElisaRSR™ IA-2 Ab Version 2 (RSR Limited, Cardiff, United Kingdom), respectively, according to the manufacturer's protocol. GADA levels ≥5.0 U/mL and IA-2A levels ≥7.5 U/mL were considered positive.

### 2.5. Statistical Analysis

To compare the sensitivity and specificity of ADAP and the currently used RIA and ELISA, we used the receiver operating characteristic (ROC) analysis with 95% CI. For perspective, the area under the curve (AUC) values from 0.5 to 0.7 for a diagnostic/prognostic test represent low accuracy, values from 0.7 to 0.9 are helpful for some purposes, and values 0.9 represent high accuracy [16].

Youden's J statistics were used to measure the ROC curve and to illustrate the effectiveness of the different autoantibodies detected by ADAP and RBA [17]. The ROC curves generated by ADAP and ELISA or RIA were considered correlated ROC curves, and nonparametric z statistics were used to compare the two methods [18]. Bland–Altman plots were used to display the degree of agreement between capillary and venous blood Ab measurements by ADAP. Bland–Altman plots are used to evaluate the agreement among two different measurement techniques, allowing the identification of any systematic difference between the measurements (i.e., proportional bias) or possible outliers. The mean difference is the estimated bias, and the SD of the differences measures the random fluctuations around this mean. If the mean value of the difference differs significantly from 0, a fixed bias is indicated. It is customary to compute 95% limits of agreement (LOA) for each comparison (average difference ± 1.96 standard deviations of the difference), which tells how far apart measurements by two methods were more likely to be for most individuals. If the differences within mean ± 1.96 SD are not clinically significant, the two methods may be used interchangeably. The difference between the methods is regressed on the average of the two methods searching for a proportional bias [19].

## 3. Results

### 3.1. ADAP in Whole Venous Blood vs. RIA and ELISA in Serum

**Figure 1:**
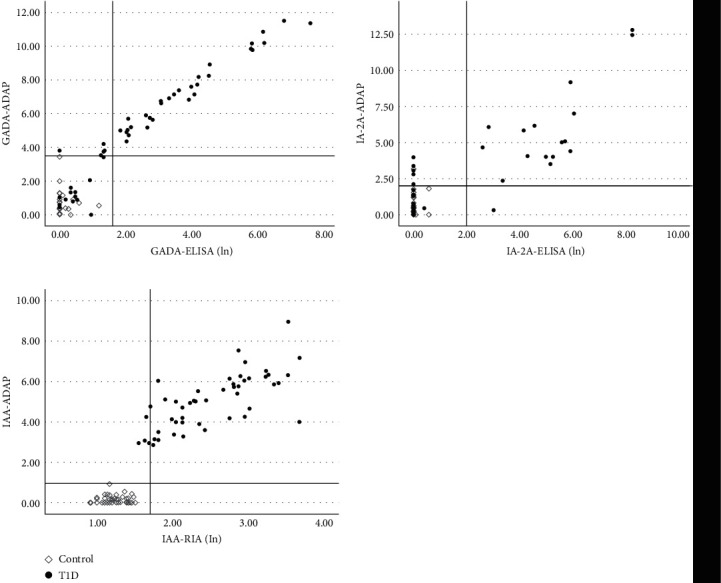
Comparison of ADAP and ELISA/RIA in venous WB for each IA. The *x*-axis displays ELISA/RIA signals in the lan scale. The *y*-axis shows the ADAP signal in *Δ*Ct. The use of logarithm was necessary as *Δ*Ct is a logarithmic parameter. The horizontal and the vertical lines indicate ADAP and ELISA/RIA cutoff thresholds, respectively.

To study the performance of the ADAP assay in venous WB, we analyzed the venous samples of the patients and controls using the ADAP assay and the currently used RIA and ELISA in serum. In the T1D group, we identified five (5.2%) patients positive for GADA in the ADAP assay and negative in ELISA, and four (4.1%) positive for IAA in the ADAP assay and negative in the RIA. In the case of IA-2A, eight (8.2%) patients with T1D were positive in the ADAP assay and negative in ELISA, one (1.0%) was negative in the ADAP assay and positive in ELISA, and one (1.0%) control was positive in ADAP and negative in ELISA (Figure 1). The agreement percentages between the assays were 94.8%, 90.8%, and 95.9% for GADA, IA-2A, and IAA, respectively. The Kappa test showed almost perfect agreement between ADAP and the currently used methods for GADA and for IAA, with *K* coefficients of 0.886 (*P*  < 0.001) and 0.918 (*P*  < 0.001), respectively. For IA-2A, the Kappa test showed substantial agreement with a *k* coefficient of 0.74 (*P*  < 0.001) (Figure 1).

**Figure 2:**
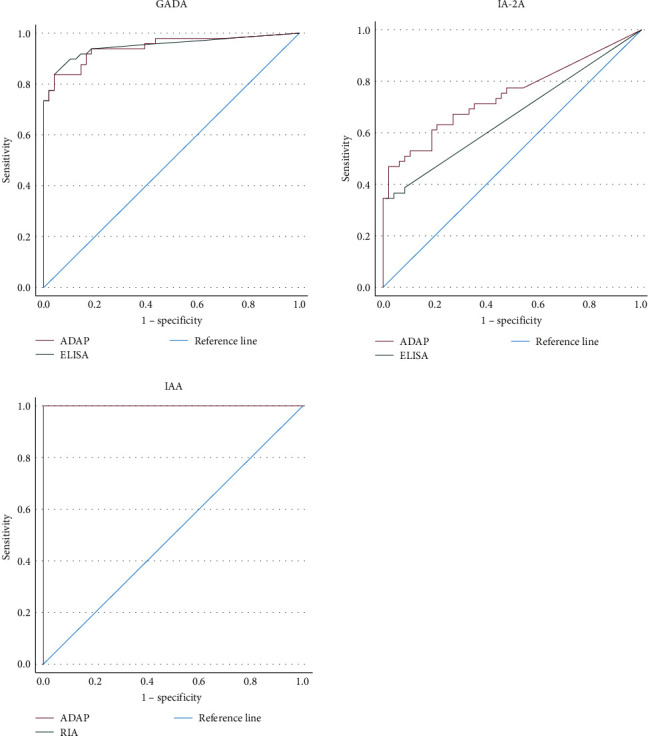
ROC curves comparing ADAP and ELISA/RIA venous WB for each IA. ADAP is in red, ELISA/RIA is in green, and the reference line is blue.

The AUC using the ROC curves was comparable between the ADAP assay in WB and currently used RIA and ELISA for all 3 IA, indicating similar clinical sensitivity and specificity: GADA 0.946 (95% CI: 0.900–0.991) vs. 0.949 (0.906–0.992), *P*=0.873; IA-2A 0.747 (0.649–0.844) vs. 0.666 (0.587–0.744), *P*=0.106; IAA 1.000 (1.000–1.000) vs. 1.000 (1.000–1.000), *P*=1.000. Nonetheless, the sensitivity and specificity for GADA and IAA using ADAP or ELISA/RIA were high while being less diagnostic for IA2A (Figure 2).

### 3.2. ADAP in Venous Whole-Blood vs. ADAP in Capillary Whole-Blood

**Figure 3:**
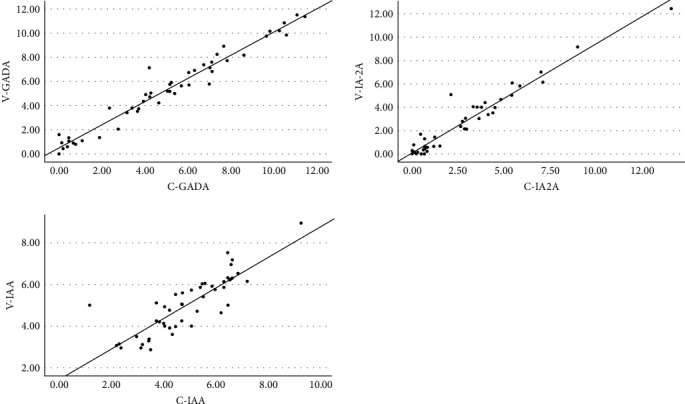
Correlation between the levels of each IA in venous and capillary WB using ADAP. V, venous; C, capillary.

**Figure 4:**
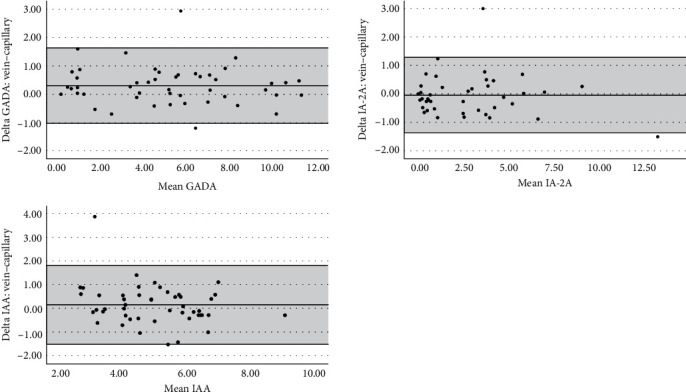
Bland–Altman's plots compare each IA in venous and capillary WB using ADAP. The shaded area represents the limits of agreement (LOA) for each comparison (average difference ± 1.96 standard deviations of the difference).

We studied the performance of the ADAP assay in venous and capillary WB of 50 T1D patients. The correlation between the levels of IA in venous and capillary WB using ADAP was *R*^2^ = 0.958 (*P* < 0.01), *R*^2^ = 0.943 (*P* < 0.01), and *R*^2^ = 0.711 (*P* < 0.01) for GADA, IA-2A and IAA, respectively (Figure 3).  Figure 4 shows Bland–Altman's plots of agreement between venous and capillary WB using ADAP measurements. Minor differences between means were observed, but no proportional bias was detected, suggesting the two methods may be used interchangeably. The mean's difference (diff) for GADA was significant. Higher levels were observed in venous compared to capillary blood, diff = 0.3 ± 0.6, 95% CI (0.11, 0.51), *P*=0.003. The mean's differences for IA2-A and IAA were nonsignificant diff = −0.05 ± 0.7 95% CI (−0.25, 0.15), *P*=0.621 and diff = 0.14 ± 0.8, 95% CI (−0.11, 0.39), *P*=0.256, respectively (Figure 4).

## 4. Discussion

The study's main goal was to validate the ability of the ADAP assay to accurately detect IAs in venous and capillary WB, proving it is suitable for a general population screening program. We found that the ADAP assay performance in detecting IA in WB is comparable to the currently used methods in serum and can be used interchangeably in venous and capillary WB.

The ADAP assay is a new platform to measure IA with high sensitivity and specificity. The assay was extensively studied in serum samples of different cohorts of T1D patients and controls, showing promising results [12–14]. Nonetheless, the assay's performance in capillary or venous WB was not studied. Thinking of a general population screening program for the detection of IA, such as the ADIR, an assay based on a small amount of WB, preferably capillary blood, would ease and reduce the costs of the screening program. Therefore, to adopt the ADAP to our program, we had to show that the assay can be done in WB, is comparable to the currently used methods, and performs interchangeably in venous and capillary blood.

Comparing ADAP to the currently used methods, the overall agreement was above 90% for all three IAs. In the samples that showed discrepant results, the ADAP assay detected additional IAs in five cases of GADA, 4 of IAA, and eight of IA2-A. One case was positive for IA-2A in ELISA and negative in ADAP. These findings align with previous studies analyzing the performance of ADAP in serum, suggesting the assay is more sensitive than RBA and, in our study, ELISA or RIA [12, 14]. ADAP's increased sensitivity stems from its unique mechanism, making the assay 100–10,000 times more analytically sensitive than conventional immunoassays [9, 10]. As in the serum, ADAP in WB could detect IA-positive patients not detected by the currently used immunoassays. Nonetheless, other assays, such as the electrochemiluminescence (ECL) assay and the luciferase immunoprecipitation system (LIPS), have also previously shown improved sensitivity and specificity for IAA and GADA compared to RIA [20–22]. Thus, it is plausible that the currently used immuno-assays are limited in detecting IA and that ADAP, like ECL and LIPS, is more precise in representing the actual IA profile.

The AUC in the ROC analysis was comparable between ADAP and the presently used ELISA and RIA for all three IAs, suggesting similar sensitivity and specificity of the assays. The ADAP assay was less diagnostic for IA-2A compared to GADA and IAA, albeit performing better than ELISA. The lower accuracy of the ADAP IA-2A assay and the discrepancy in the results compared to conventional immunoassays were demonstrated in previous studies [12, 14]. IA-2 is a protein tyrosine phosphatase primarily localized on secretory granules within the beta-cell. Several phosphatase domains have been suggested as epitopes for IA-2 immunoglobulins [23]. Thus, it is plausible that ADAP or the conventional assays do not detect some IA-2 immunoglobulins and that different assays detect distinct IA-2 antibodies, causing this discrepancy. Nonetheless, as previously mentioned, the ADAP assay detected more IA-2A positive cases among T1D patients, suggesting it is more sensitive than ELISA.

Capillary blood is a more desirable matrix than venous WB or serum for general-population screening. It can be easily collected without phlebotomy and bypass the need for serum separation, thereby utilizing a small amount of blood and simplifying the analysis process. Our results demonstrate that the ADAP assay is comparable in venous and capillary blood WB. This was shown in the correlation analyses and Bland–Altman's analysis. A mean's difference was observed for GADA in Bland–Altman's analysis, showing higher levels in venous compared to capillary blood. However, no proportional bias was detected between capillary and venous blood, suggesting that the two methods may be used interchangeably with proper adjustments. Although the difference we observed between the means is minimal and within the standard deviation of the assay, our findings imply that the threshold of GADA positivity should be adjusted according to the source of the sample.

For the needs of our screening program, the study's main goal was to validate the ability of the ADAP assay to detect IAs in capillary WB accurately. We showed that the assay is comparable in capillary and venous WB. Perhaps a study design that included analyzing ADAP in the serum in addition to venous WB would have been more appropriate to prove our point. However, as the assay was previously evaluated in the serum, and our findings in WB align with these findings, we find the assay in capillary blood suitable for our screening program [12–14].

Our study has some limitations. We did not evaluate ADAP in the general population, and our findings on a relatively small cohort of children with T1D treated with insulin do not stand alone. The IA profile in the general population is different than that of children with T1D treated with insulin, and therefore, our findings may be biased by the patient selection. However, with the previous studies analyzing the ADAP assay in serum samples, our results suggest that ADAP may be a proper alternative for a general population screening program as a first-line screen or a confirmatory test. Moreover, the study only evaluated the performance of the ADAP assay in detecting IAs in WB and did not assess its clinical utility in predicting T1D development or progression. Further studies with larger sample sizes and long-term follow-up are needed to validate the clinical utility of the ADAP assay in general population screening programs.

In conclusion, the ADAP assay is reliable in detecting IA in venous and capillary WB samples with comparable performance to standard RIA and ELISA. These findings open avenues for widespread use of the ADAP assay as a first-line screen or a confirmatory test in future general population screening programs aiming to detect children at risk of developing T1D.

## Data Availability

Data are available upon request from the corresponding other with legal approval of the institutional ethics committee.
